# The economic value of vaccination: why prevention is wealth

**DOI:** 10.3402/jmahp.v3.29284

**Published:** 2015-08-12

**Authors:** Vanessa Rémy, Nathalie Largeron, Sibilia Quilici, Stuart Carroll

**Affiliations:** 1 Sanofi Pasteur MSD, Lyon, France; 2 Sanofi Pasteur MSD, Maidenhead, United Kingdom

Theoretical and empirical evidence has demonstrated that health has a major role to play as a driver for economic growth. Improving health outcomes can have a positive impact on economic outcomes and societal well-being, for example, through longer working lives, higher productivity, improved educational outcomes, social inclusion, and reduced healthcare costs (1).

The economic crisis that started in Europe in 2008 has put tremendous pressure on national budgets, leading to arbitrary cuts with important consequences for healthcare systems and the health of European citizens. It has been predicted that the number of people aged ≥65 years will almost double, and those aged ≥80 years will triple, by 2060 in the European Union (EU) (2). The combination of the difficult economic situation and the demographic pressure results in important challenges to meet the higher demand for healthcare and to adapt healthcare systems to the needs of an ageing population while keeping them sustainable.

Key opinion leaders, scientific experts, as well as governments and national budget holders face the same dilemma: how to spend the limited financial resources dedicated to healthcare more efficiently for the benefit of the population. One approach to do this, while improving the health of Europeans, is to concentrate our efforts on keeping people healthy, rather than waiting to treat them once they become ill. Prevention is one of the best means of helping Europeans to live healthier and longer, thereby increasing European productivity.

**Fig. 1:**
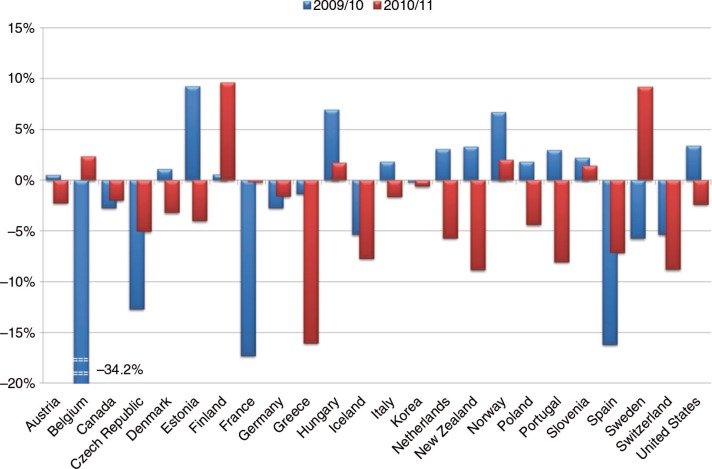
Evolution of healthcare prevention budgets in Europe (3).

In Europe, the national budgets allocated to healthcare represent an average of 9.0% of the gross domestic product (GDP), although on average, only 3% of this budget is dedicated to prevention (3). The preventative healthcare budget is allocated to diverse areas such as smoking cessation, reduction of alcoholism, improved nutrition, encouraging physical activity, and higher uptake of vaccinations. This budget has decreased in many EU countries over recent years, except in 2009 due to the pandemic flu crisis (Fig. 1) (3). Generally, preventative programmes are most vulnerable to budget cuts and restrictions since their benefits are not always immediately identifiable. These cuts often have a short-term focus and do not affect directly identifiable patients, but they do, however, affect future public health. Thus, new healthcare models are needed to change the focus of current systems from illness management to healthcare management integrating cost-effective preventative interventions.

Although a minor fraction of the healthcare budget is allocated to vaccination programmes (e.g., equivalent to about 0.3% of healthcare expenditure in France), they have a central role in prevention policies (4). The recent European Council conclusions on ‘vaccination as an effective tool in public health’ highlighted that an evidence-based, cost-effective, safe, and efficient vaccination system is an integral part of a well-functioning health system (5). Vaccines are among the most successful and cost-effective public health tools for preventing diseases and death. They have led to the eradication of smallpox, the elimination of polio from most continents, and the control of other diseases, including diphtheria, tetanus, pertussis, rubella, and hepatitis B. The impact of vaccines can be measured not just in terms of public health, but also in economic terms: reducing healthcare costs, decreasing lost labour force productivity, and contributing to social and economic development (6, 7).

In December 2014, The Council of European Ministers of Health updated the Europe 2020 Strategy, to acknowledge the importance of investment in health as a contributor to economic growth, highlighting the role of preventative actions, including vaccination against communicable diseases, in strengthening cost-effectiveness (8). Vaccination and the vaccine industry are undeniably key contributors to the smart, sustainable, and inclusive growth objectives for Europe 2020. With about 80% of the world's vaccine production occurring in Europe, the vaccine industry represents a key contributor to employment and productivity with a higher than average proportion of employees having tertiary-level education. Vaccine-related research and development is also one of the highest (15% of sales) among all industries (9). Lastly, infectious disease control and vaccination coverage may act as an effective safeguard against poverty and health inequalities.

Governments and policy makers will need to acknowledge that prevention through vaccination involves low levels of investment relative to the substantial benefits procured for European citizens and the European economy. Several countries, such as the United States, Canada, and Australia, have begun to consider vaccination as pivotal in their prevention programmes. Taking into account the full economic benefits of vaccination would allow positioning prevention as one of the best ways to identify efficiency gains.

This special issue in the *Journal of Market Access and Health Policy* is made up of a series of seven articles that aim to provide policy makers with robust evidence on the economic benefits of vaccination in Europe, from different angles and perspectives. We acknowledge the limitations of this series in focusing on the benefits of vaccination from an economic perspective and, thus, may not fully address the potential drawbacks of vaccines, in particular from a clinical perspective. The objectives of this special issue are threefold:

- To demonstrate the full economic value of vaccination based on published examples;
- To describe the contribution that vaccination can make to healthcare systems’ sustainability and efficiency and also to the wider economy;
- To launch a call for action for developing and implementing tools that allow the full economic value of vaccination to be taken into consideration.

Together, the authors would like to make a call to each country, each region, and each health constituency to appreciate the level of budget that they allocate to this important public health intervention. Indeed, there is a strong case for renewed European commitment to vaccination that requires action on several fronts:

- ***Shift in mind and in budget***: Given the undeniable importance of vaccination for public health, there is a need to secure an appropriate level of budget to guarantee populations’ access to vaccination;
- ***Shift in communication***: There is a need to communicate clearer and more compelling messages about the value of vaccination to governments, policy makers, as well as healthcare professionals and the lay public;
- ***Shift in vaccines evaluation***: There is a need to consider the global economic benefits from vaccination. Economic evaluations should move beyond assessing only the effect of vaccination on health and medical costs at the individual level, and address the broader economic value to society.
